# Prediction of uncomplicated pregnancies in obese women: a prospective multicentre study

**DOI:** 10.1186/s12916-017-0956-8

**Published:** 2017-11-03

**Authors:** Matias C. Vieira, Sara L. White, Nashita Patel, Paul T. Seed, Annette L. Briley, Jane Sandall, Paul Welsh, Naveed Sattar, Scott M. Nelson, Debbie A. Lawlor, Lucilla Poston, Dharmintra Pasupathy, on behalf of the UPBEAT Consortium, Lucilla Poston, Andrew Shennan, Annette Briley, Claire Singh, Paul Seed, Jane Sandall, Thomas Sanders, Nashita Patel, Angela Flynn, Shirlene Badger, Suzanne Barr, Bridget Holmes, Louise Goff, Clare Hunt, Judy Filmer, Jeni Fetherstone, Laura Scholtz, Hayley Tarft, Anna Lucas, Tsigerada Tekletdadik, Deborah Ricketts, Carolyn Gill, Alex Seroge Ignatian, Catherine Boylen, Funso Adegoke, Elodie Lawley, James Butler, Rahat Maitland, Matias Vieira, Dharmintra Pasupathy, Eugene Oteng-Ntim, Nina Khazaezadeh, Jill Demilew, Sile O’Connor, Yvonne Evans, Susan O’Donnell, Ari de la Llera, Georgina Gutzwiller, Linda Hagg, Stephen Robson, Ruth Bell, Louise Hayes, Tarja Kinnunen, Catherine McParlin, Nicola Miller, Alison Kimber, Jill Riches, Carly Allen, Claire Boag, Fiona Campbell, Andrea Fenn, Sarah Ritson, Alison Rennie, Robin Durkin, Gayle Gills, Roger Carr, Scott Nelson, Naveed Sattar, Therese McSorley, Hilary Alba, Kirsteen Paterson, Janet Johnston, Suzanne Clements, Maxine Fernon, Savannah Bett, Laura Rooney, Sinead Miller, Paul Welsh, Lynn Cherry, Melissa Whitworth, Natalie Patterson, Sarah Lee, Rachel Grimshaw, Christine Hughes, Jay Brown, Kim Hinshaw, Gillian Campbell, Joanne Knight, Diane Farrar, Vicky Jones, Gillian Butterfield, Jennifer Syson, Jennifer Eadle, Dawn Wood, Merane Todd, Asma Khalil, Deborah Brown, Paola Fernandez, Emma Cousins, Melody Smith, Jane Wardle, Helen Croker, Laura Broomfield, Keith Godfrey, Sian Robinson, Sarah Canadine, Lynne Greenwood, Catherine Nelson-Piercy, Stephanie Amiel, Gail Goldberg, Daghni Rajasingham, Penny Jackson, Sara Kenyon, Patrick Catalano

**Affiliations:** 1 Department of Women and Children’s Health, School of Life Course Sciences, Faculty of Life Sciences and Medicine, King’s College London, London, SE1 7EH UK; 2 Núcleo de Formação Específica em Ginecologia e Obstetrícia, Escola de Medicina, Pontifícia Universidade Católica do Rio Grande do Sul, Porto Alegre, 90610-000 Brazil; 3 NIHR Biomedical Research Centre at Guy’s and St Thomas’ NHS Foundation Trust and King’s College London, London, SE1 7EH UK; 4 Institute of Cardiovascular and Medical Sciences, University of Glasgow, Glasgow, G12 8TA UK; 5 School of Medicine, University of Glasgow, Glasgow, G4 0SF UK; 6 MRC Integrative Epidemiology Unit and School of Social and Community Medicine, University of Bristol, Bristol, BS8 2BN UK; 7 NIHR Biomedical Research Centre at University Hospitals Bristol NHS Foundation Trust and University of Bristol, Bristol, BS8 2BN UK; 8 Department of Women and Children’s Health, Women’s Health Academic Centre KHP, St. Thomas’ Hospital, Westminster Bridge Road, 10th Floor North Wing, London, SE1 7EH UK

**Keywords:** Obesity, Prediction, Uncomplicated pregnancy, Birth, Pregnancy outcome, Risk stratification

## Abstract

**Background:**

All obese pregnant women are considered at equal high risk with respect to complications in pregnancy and birth, and are commonly managed through resource-intensive care pathways. However, the identification of maternal characteristics associated with normal pregnancy outcomes could assist in the management of these pregnancies. The present study aims to identify the factors associated with uncomplicated pregnancy and birth in obese women, and to assess their predictive performance.

**Methods:**

Data form obese women (BMI ≥ 30 kg/m^2^) with singleton pregnancies included in the UPBEAT trial were used in this analysis. Multivariable logistic regression was used to identify sociodemographic, clinical and biochemical factors at 15^+0^ to 18^+6^ weeks’ gestation associated with uncomplicated pregnancy and birth, defined as delivery of a term live-born infant without antenatal or labour complications. Predictive performance was assessed using area under the receiver operating characteristic curve (AUROC). Internal validation and calibration were also performed. Women were divided into fifths of risk and pregnancy outcomes were compared between groups. Sensitivity, specificity, and positive and negative predictive values were calculated using the upper fifth as the positive screening group.

**Results:**

Amongst 1409 participants (BMI 36.4, SD 4.8 kg/m^2^), the prevalence of uncomplicated pregnancy and birth was 36% (505/1409). Multiparity and increased plasma adiponectin, maternal age, systolic blood pressure and HbA1c were independently associated with uncomplicated pregnancy and birth. These factors achieved an AUROC of 0.72 (0.68–0.76) and the model was well calibrated. Prevalence of gestational diabetes, preeclampsia and other hypertensive disorders, preterm birth, and postpartum haemorrhage decreased whereas spontaneous vaginal delivery increased across the fifths of increasing predicted risk of uncomplicated pregnancy and birth. Sensitivity, specificity, and positive and negative predictive values were 38%, 89%, 63% and 74%, respectively. A simpler model including clinical factors only (no biomarkers) achieved an AUROC of 0.68 (0.65–0.71), with sensitivity, specificity, and positive and negative predictive values of 31%, 86%, 56% and 69%, respectively.

**Conclusion:**

Clinical factors and biomarkers can be used to help stratify pregnancy and delivery risk amongst obese pregnant women. Further studies are needed to explore alternative pathways of care for obese women demonstrating different risk profiles for uncomplicated pregnancy and birth.

**Electronic supplementary material:**

The online version of this article (doi:10.1186/s12916-017-0956-8) contains supplementary material, which is available to authorized users.

## Background

Maternal obesity is associated with increased risk of maternal and perinatal mortality and morbidity, including gestational diabetes mellitus (GDM), preeclampsia and birth complications [1]. In common with global trends of obesity in the non-pregnant population, the prevalence of maternal obesity is increasing, with one in four women of reproductive age in the UK being classed as obese [1, 2]. The NHS costs of care and management of pregnancy complications in obese women have been estimated at an additional £1171 (37%) per pregnancy compared to women of normal body mass index (BMI) [3].

A key strategy of the UK 2016 Maternity Transformation Programme is the evidence-based provision of antenatal care that recognises different levels of risk of adverse outcomes between different groups of women [4]. The UK National Maternity Review, ‘Better Births’ [5], notes that a simple dichotomy of ‘high risk’ or ‘no risk’ is too simplistic, and women are requesting more detailed information about their risks. In this context, defining all obese pregnant women as ‘high risk’ may not always ensure provision of the most effective care nor a choice of care pathway. We have previously suggested that maternity care might benefit from a shift away from the current focus on complications towards the pursuit of uncomplicated pregnancy [6]. The identification of maternal characteristics associated with normal pregnancy outcomes in obese women and the combining of these factors in a risk stratification algorithm could help inform women’s decision-making with regards to the management of their pregnancies as well as the provision and allocation of resources for pregnancy care for obese women.

The aim of the present study is to identify factors in the early second trimester associated with subsequent uncomplicated pregnancy and birth in obese women and to assess their predictive performance.

## Methods

The UK Pregnancies Better Eating and Activity Trial (UPBEAT; ISCRTN89971375) was a multicentre, randomised controlled trial of a complex behavioural intervention of diet and physical activity advice versus standard antenatal care in obese pregnant women to prevent GDM and delivery of large for gestational age infants. The study involved eight centres located in London (three centres), Bradford, Glasgow, Manchester, Newcastle, and Sunderland. Regulatory approvals were obtained from the UK research ethics committee (UK integrated research application system, reference 09/H0802/5) and local research and development departments in participating centres. All women provided written informed consent prior to entering the study.

UPBEAT recruited 1555 obese women (BMI ≥ 30 kg/m^2^), aged 16 years or older, and with a singleton pregnancy between 15^+0^ and 18^+6^ weeks’ gestation. Women were recruited from March 2009 to June 2014. Exclusion criteria were multiple pregnancy, current use of metformin, unwilling or unable to provide written informed consent, or underlying disorders (including a pre-pregnancy diagnosis of essential hypertension, diabetes, renal disease, systemic lupus erythematosus, antiphospholipid syndrome, sickle cell disease, thalassaemia, coeliac disease, thyroid disease or current psychosis). Extensive data were collected on sociodemographic and clinical characteristics, and anthropometric measures and blood samples were also obtained. Women were followed up at 27^+0^ to 28^+6^ and 34^+0^ to 35^+6^ weeks’ gestation, at delivery and at 6 months postpartum [7, 8]. Except for the UPBEAT behavioural intervention, routine antenatal care according to UK and local practice was provided to all women in the study.

For the purpose of this study, women with missing information for pregnancy outcomes were excluded. The UPBEAT intervention was not associated with an effect on the primary outcomes or any relevant pregnancy outcome [8], and therefore the study population was treated as a cohort for the purpose of this analysis [9].

### Outcomes

The outcome of interest was an uncomplicated pregnancy and birth defined in this study as a pregnancy without any antenatal or labour complications following recruitment at 15^+0^ to 18^+6^ weeks’ gestation. Antenatal complications were late miscarriage, preterm birth (before 37 weeks’ gestation), GDM, preeclampsia, hypertensive disorders of pregnancy, antepartum haemorrhage, placental abruption, venous thromboembolism, delivery of a small for gestational age infant (birthweight below the 10th customised centile [10]) and stillbirth (after 20 weeks’ gestation). Labour complications were instrumental vaginal birth, emergency caesarean section, postpartum haemorrhage (above 1000 mL), Apgar score < 7 at 5 minutes, neonatal intensive care unit admission and neonatal death. Preeclampsia was defined as blood pressure ≥ 140/90 mmHg associated with proteinuria of ≥ 300 mg/24 h, or a spot urine protein:creatinine ratio of ≥ 30 mg/mmol creatinine, or urine dipstick protein ≥ 2+ [11], and a GDM diagnosis was based on a 75 g oral glucose tolerance test (OGTT) using the International Association of Diabetes and Pregnancy Study Groups (IADPSG) criteria [12]. Universal OGTT was part of the study protocol but, where not performed, a clinical diagnosis of GDM was used.

### Potential predictors of uncomplicated pregnancy and birth

Factors from early pregnancy (15^+0^ to 18^+6^ weeks’ gestation) were selected on the basis of known associations with one or more pregnancy complications. The sociodemographic characteristics and clinical factors explored were maternal age, ethnicity (white, black, Asian or other), adjusted index of multiple deprivation, BMI, parity, previous history of GDM or preeclampsia, smoking, history of threatened miscarriage in the index pregnancy, systolic blood pressure (SBP) and maternal anthropometry. Maternal anthropometric measures included were mid-arm circumference and sum of skinfold thickness (triceps, biceps, subscapular and suprailiac). Blood pressure was recorded using the pregnancy validated Microlife BP3BT0-A blood pressure monitor (Microlife, Widnau, Switzerland) and maternal skinfold thicknesses (triceps, biceps, suprailiac and subscapular) were measured in triplicate, using Harpenden skinfold Calipers (Holtain Ltd, Felin-y-Gigfran, Crosswell, UK) [13].

A total of 19 selected biomarkers measured in blood samples obtained at 15^+0^ to 18^+6^ weeks’ gestation were assessed. The biomarkers measured in blood samples were markers of glucose homeostasis (haemoglobin A1c (HbA1c), fructosamine, insulin and C-peptide), adipokines (adiponectin and leptin), inflammatory and endothelial markers (interleukin-6, high sensitivity C-reactive protein, and t-PA antigen), lipids (triglycerides, total cholesterol, LDL cholesterol and HDL cholesterol), liver-associated markers (aspartate aminotransferase, alanine aminotransferase, gamma-glutamyl transferase (gGT), sex hormone binding globulin and ferritin) and vitamin D. Analytical methods for these biomarkers are shown in Additional file 1.

### Statistical analysis

All participants with complete pregnancy outcome data were included in the analysis. To ensure that the study population was representative of the overall UPBEAT participants we compared demographic characteristics between women included and excluded from this analysis. Missing data on potential predictors were minimal (< 2%), except for biomarkers, as blood samples were not available for 27% of women (286/1409). Of those with blood samples taken, biomarker missing data were minimal (< 2%), except for HbA1c (5.7%; 59/1023). Biomarkers were assessed for normality and variation with gestational age. None of the biomarkers showed variation with gestational age at measurement and biomarkers with highly skewed distributions were transformed into log_2_ (insulin, C-peptide, adiponectin, leptin, interleukin-6, high sensitivity C-reactive protein, t-PA antigen, triglycerides, HDL cholesterol, aspartate aminotransferase, alanine aminotransferase, gGT, ferritin and vitamin D), so that odds ratios (ORs) showed the effect associated with doubling the concentration. Ethnicity was transformed into a binary (white and non-white) variable for analysis to reduce the degrees of freedom. The index of multiple deprivation was categorised into fifths for a clearer interpretation. Continuous data were summarised by mean (standard deviation) and categorical data reported as number (percentage). Comparisons between groups were performed using a *t*-test or χ^2^ test, as appropriate. Univariable logistic regression was used to estimate the effect size for each factor. Clinical factors with a *P* value less than 0.05 in group comparison were included in multivariable analyses. Biomarkers with a statistically significant association following Bonferroni correction for 19 tests (*P* < 0.0026) were included in multivariable analyses.

Backward stepwise logistic regression was then used to identify factors that were independently associated with uncomplicated pregnancies and birth [14]. One multivariable model was developed to include only clinical factors, and a second model included both clinical factors and biomarkers. A sensitivity analysis was performed to assess if the reduction in the sample size for the assessment of biomarkers had any effect on the size of the association between clinical factors and outcome. The outcome of this study was a normal event instead of an adverse outcome, factors with an OR smaller than one were considered predictors of an adverse outcome, whereas those with an OR greater than one were considered to predict increased likelihood of an uncomplicated pregnancy. The overall accuracy of the final multivariable model to discriminate between uncomplicated and complicated pregnancy was assessed using the area under the receiver operating characteristic curve (AUROC). Internal cross-validation using a 10-fold split in the dataset was performed [15]. Calibration was assessed by comparing observed to predicted levels of uncomplicated pregnancy and birth within tenths of predicted risk and a Hosmer–Lemeshow test was performed. Women were then divided into fifths of predicted risk and pregnancy outcomes were compared between the fifths. Finally, the upper fifth of predicted levels of uncomplicated pregnancy and birth was considered a positive screening group and diagnostic test performance was assessed (sensitivity, specificity, positive predictive value and negative predictive value). All statistical analyses were performed using Stata software, version 14.1 (StataCorp, College Station, Texas). This study was reported in accordance with the TRIPOD guideline for reporting the development of multivariable prediction models [16].

## Results

**Fig. 1:**
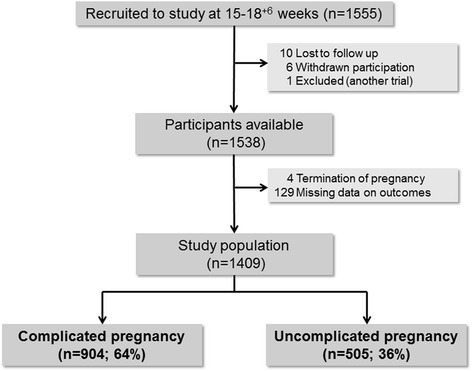
Study population

The UPBEAT trial recruited 1555 obese women between 15^+0^ and 18^+6^ weeks’ gestation. In this analysis, the study population comprised 1409 women with complete outcome data (Fig. 1) and there were no obvious differences in demographic characteristics between our study population and UPBEAT participants excluded from this analysis. Additional file 2 describes these comparisons. The prevalence of uncomplicated pregnancy and birth in the study population was 36% (505/1409).

Amongst women with complications, 38% (342/904) had antenatal complications alone, 32% (287/904) had labour complications alone and 30% (275/904) experienced both. Women who experienced both antenatal and labour complications accounted for 20% (275/1409) of the whole study population (Additional file 3 shows this distribution). The prevalence of each pregnancy complication is described in Table 1. GDM was the leading antenatal complication and emergency caesarean section was the most prevalent labour complication.

**Table 1:** Prevalence of each pregnancy complication

	N (*n* = 1409)	Percent
Antenatal complications		
Miscarriage	8	0.6
Preterm birth	77	5.5
Gestational diabetes	334	23.7
Preeclampsia	53	3.8
Other hypertensive disorders	83	5.9
Antepartum haemorrhage	54	3.8
Placental abruption	9	0.6
Venous thromboembolism	4	0.3
Small for gestational age	162	11.5
Stillbirth	7	0.5
Labour complications		
Instrumental delivery	165	11.7
Emergency caesarean section	237	16.8
Postpartum haemorrhage	186	13.2
Apgar < 7 at 5 min	28	2.0
Neonatal intensive care	113	8.0
Neonatal death	4	0.3

The sociodemographic characteristics of women with and without complications in pregnancy are described in Table 2, and the descriptive statistics for potential predictors of uncomplicated pregnancy and birth are shown in Additional file 4.

**Table 2:** Sociodemographic characteristics in pregnancies with and without complications in pregnancy and/or birth

	Complicated pregnancy, Mean (SD) or n (%)	Uncomplicated pregnancy, Mean (SD) or n (%)
Age	30.6 (5.4)	30.0 (5.7)
Body mass index	36.6 (5.2)	35.9 (4.1)
Ethnicity		
White	567/904 (62.7)	319/505 (63.2)
Black	224/904 (24.8)	132/505 (26.1)
Asian	55/904 (6.1)	31/505 (6.1)
Other	58/904 (6.4)	23/505 (4.6)
Multiparous	416/904 (46.0)	370/505 (73.3)
Previous history of GDM	22/416 (5.3)	6/370 (1.6)
Previous history of PE	37/416 (8.9)	25/370 (6.8)
IMD fifths		
1 (least deprived)	33/899 (3.7)	19/504 (3.8)
2	62/899 (6.9)	32/504 (6.4)
3	95/899 (10.6)	61/504 (12.1)
4	316/899 (35.2)	164/504 (32.5)
5 (most deprived)	393/899 (43.7)	228/504 (45.2)
Current smoker	66/904 (7.3)	32/505 (6.3)

*GDM* gestational diabetes mellitus, *IMD* index of multiple deprivation, *PE* preeclampsia

**Fig. 2:**
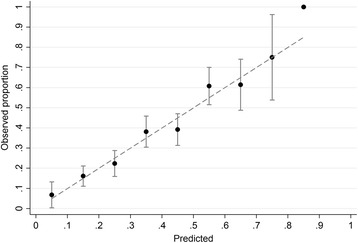
Calibration of the full prediction model for uncomplicated pregnancies in obese women

In univariable analyses, multiparity was associated with increased odds of an uncomplicated pregnancy and birth, and factors associated with a reduced odds were higher maternal age, SBP, BMI, mid-arm circumference and sum of skinfolds (Table 3). A previous history of either GDM or preeclampsia was also associated with reduced odds of uncomplicated pregnancy and birth. Higher concentrations of adiponectin were associated with greater odds of uncomplicated pregnancy and birth, while reduced odds were observed for higher HbA1c, insulin, sex hormone binding globulin and gGT. In multivariable analysis, multiparity, SBP, adiponectin and HbA1c were independently associated with uncomplicated pregnancy and birth (Table 3). Using these factors from multivariable analysis, the algorithm achieved an AUROC of 0.73 (0.69–0.76) (Table 3). Model performance was confirmed by internal validation with an AUROC of 0.72 (0.68–0.76). The model also had good calibration as demonstrated by the similarity of predicted and observed proportions of uncomplicated pregnancies and births across tenths of predicted risk (Fig. 2; Hosmer–Lemeshow *P* = 0.54).

**Table 3:** Factors associated with uncomplicated pregnancies and birth, and their predictive performance

	Univariable^a^, OR (95% CI)	Multivariable (Clinical factors only), OR (95% CI), *n* = 1370	Multivariable (All factors), OR (95% CI), *n* = 907
Maternal age (per 5 year), *n* = 1409	0.90 (0.82–1.00)	0.79 (0.71–0.88)	0.81 (0.71–0.93)
Multiparous, *n* = 1409	3.22 (2.54–4.07)	3.54 (2.75–4.55)	4.23 (3.04–5.88)
Previous history of GDM or PE, *n* = 786	0.58 (0.36–0.91)		
SBP (per 10 mmHg), *n* = 1387	0.77 (0.69–0.85)	0.82 (0.73–0.91)	0.84 (0.73–0.97)
BMI (per 5 kg/m^2^), *n* = 1409	0.84 (0.74–0.94)	0.85 (0.74–0.97)	
Mid-arm circumference (per 1 cm) *n* = 1398	0.96 (0.94–0.99)		
Sum of skinfolds (per 1 cm), *n* = 1390	0.94 (0.91–0.98)		
Biomarkers			
HbA1c (per 1 mmol/mol), *n* = 949	0.90 (0.87–0.94)		0.90 (0.86–0.94)
Insulin (per doubling, log_2_ of mU/L), *n* = 1017	0.86 (0.78–0.94)		
Adiponectin (per doubling, log_2_ of μg/mL), *n* = 1013	1.34 (1.16–1.56)		1.40 (1.18–1.66)
gGT (per doubling, log_2_ of U/L), *n* = 1015	0.78 (0.67–0.89)		
SHBG (per 1 nmol/L), *n* = 1004	1.00 (1.00–1.00)		
AUROC		0.69 (0.66–0.71)	0.73 (0.69–0.76)
Internal validation AUROC		0.68 (0.65–0.71)	0.72 (0.68–0.76)

^a^In univariable analyses numbers vary depending on missing data and are given in the first column

*AUROC* area under the receiver operating characteristic curve, *BMI* body mass index, *GDM* gestational diabetes mellitus, *gGT* gamma-glutamyl transferase, *HbA1c* haemoglobin A1c, *PE* preeclampsia, *SBP* systolic blood pressure, *SHBG* sex hormone binding globulin

Women were then divided in fifths of predicted risk and the prevalence of each adverse outcome in the fifths is described in Table 4. With an increasing predicted chance of uncomplicated pregnancies and birth, a trend of reduced prevalence of antenatal (preterm delivery, gestational diabetes, preeclampsia and other hypertensive disorders) and labour complications (caesarean section and postpartum haemorrhage) were observed. Sensitivity, specificity, and positive and negative predictive value of the upper (5th) fifth (compared with all other women) for an uncomplicated pregnancy and birth were 38% (32–43%), 89% (86–91%), 63% (55–70%) and 74% (71–77%), respectively. Positive likelihood ratio and negative likelihood ratio were also calculated (3.3 and 0.7, respectively).

**Table 4:** Pregnancy outcomes according to predicted chance in fifths of uncomplicated pregnancy and birth using the full model

	Least likely to have an uncomplicated pregnancy and birth			Most likely to have an uncomplicated pregnancy and birth		
	1st fifth(*n* = 181)	2nd fifth(*n* = 181)	3rd fifth(*n* = 182)	4th fifth(*n* = 181)	5th fifth(*n* = 182)	*P* value
Uncomplicated pregnancy (%)	22 (12.2)	34 (18.8)	58 (31.9)	75 (41.4)	114 (62.6)	< 0.001
Antenatal outcomes (%)						
Miscarriage^a^	2 (1.1)	2 (1.1)	1 (0.5)	3 (1.7)	0 (0)	0.51
Preterm delivery	11 (6.1)	16 (8.9)	6 (3.3)	7 (3.9)	5 (2.7)	0.04
Gestational diabetes	77 (43)	50 (27.9)	57 (31.5)	35 (19.7)	29 (15.9)	< 0.001
Preeclampsia	12 (6.7)	13 (7.3)	6 (3.3)	3 (1.7)	6 (3.3)	0.04
Other hypertensive disorders	16 (8.9)	12 (6.7)	13 (7.2)	9 (5.1)	2 (1.1)	0.02
Antepartum haemorrhage	11 (6.1)	4 (2.2)	8 (4.4)	12 (6.7)	3 (1.6)	0.06
Placental abruption	2 (1.1)	0 (0)	1 (0.6)	1 (0.6)	2 (1.1)	0.68
Venous thromboembolism	0 (0)	1 (0.6)	0 (0)	0 (0)	0 (0)	0.40
Small for gestational age	26 (14.5)	31 (17.3)	18 (9.9)	18 (10.1)	18 (9.9)	0.10
Large for gestational age	16 (8.9)	11 (6.1)	18 (9.9)	17 (9.6)	18 (9.9)	0.69
Stillbirth	2 (1.1)	0 (0)	1 (0.6)	1 (0.6)	1 (0.5)	0.73
Labour outcomes (%)						
Induction of labour	95 (52.0)	76 (42.5)	60 (33.1)	45 (25.3)	51 (28.0)	< 0.001
Elective caesarean section	41 (22.9)	22 (12.3)	34 (18.8)	41 (23.0)	44 (24.2)	0.03
Mode of delivery (in labour)						
Spontaneous vaginal	56 (40.6)	76 (48.4)	94 (63.9)	105 (76.6)	127 (92.0)	< 0.001
Instrumental delivery	27 (19.6)	35 (22.3)	21 (14.3)	16 (11.7)	3 (2.2)	< 0.001
Emergency CS	55 (39.9)	46 (29.3)	32 (21.8)	16 (11.7)	8 (5.8)	< 0.001
Postpartum haemorrhage	44 (24.6)	28 (15.6)	27 (14.9)	20 (11.2)	16 (8.8)	< 0.001
Apgar < 7 at 5 min	6 (3.4)	4 (2.2)	2 (1.1)	4 (2.2)	3 (1.6)	0.66
NICU admission	19 (10.6)	20 (11.2)	15 (8.3)	12 (6.7)	10 (5.5)	0.24
Neonatal death	1 (0.6)	1 (0.6)	0 (0)	0 (0)	1 (0.5)	0.74

^a^Women with miscarriage were not included in the analysis of other outcomes

*CS* caesarean section, *NICU* neonatal intensive care unit

The model including only clinical factors (AUROC of 0.68, 0.65–0.71) was also used to explore the prevalence of each adverse outcome in each fifth of predicted risk (Table 5). Diagnostic test performance was also calculated for this model. Sensitivity, specificity, and positive and negative predictive values of the upper fifth were 31% (27–36%), 86% (84–89%), 56% (50–62%) and 69% (66–72%), respectively. The positive and negative likelihood ratios were 2.3 and 0.8, respectively. Additional file 5 describes the fifths of predicted risk for an alternative model including clinical factors and HbA1c (AUROC 0.71, 0.66–0.75). This post-hoc analysis was performed as HbA1c is readily available in clinical practice in most countries, while adiponectin is more often available in research settings. Sensitivity, specificity, and positive and negative predictive values of the upper fifth using this alternative model were 33% (29–40%), 87% (84–90%), 57% (49–64%) and 72% (69–76%), respectively.

**Table 5:** Pregnancy outcomes according to predicted chance in fifths of uncomplicated pregnancy and birth using the clinical model

	Least likely to have an uncomplicated pregnancy and birth			Most likely to have an uncomplicated pregnancy and birth		
	1st fifth(*n* = 181)	2nd fifth(*n* = 181)	3rd fifth(*n* = 182)	4th fifth(*n* = 181)	5th fifth(*n* = 182)	*P* value
Uncomplicated pregnancy (%)	47 (17.2)	67 (24.5)	98 (35.8)	126 (46)	154 (56.2)	< 0.001
Antenatal outcomes (%)						
Miscarriage^a^	2 (0.7)	1 (0.4)	2 (0.7)	2 (0.7)	1 (0.4)	0.94
Preterm delivery	13 (4.8)	23 (8.4)	8 (2.9)	17 (6.3)	14 (5.1)	0.07
Gestational diabetes	82 (30.1)	59 (21.6)	73 (26.8)	71 (26.1)	39 (14.3)	< 0.001
Preeclampsia	16 (5.9)	17 (6.2)	8 (2.9)	6 (2.2)	4 (1.5)	0.006
Other hypertensive disorders	37 (13.6)	9 (3.3)	19 (7.0)	12 (4.4)	5 (1.8)	< 0.001
Antepartum haemorrhage	15 (5.5)	8 (2.9)	9 (3.3)	14 (5.1)	7 (2.6)	0.26
Placental abruption	2 (0.7)	2 (0.7)	2 (0.7)	3 (1.1)	0 (0)	0.61
Venous thromboembolism	2 (0.7)	1 (0.4)	0 (0)	0 (0)	0 (0)	0.25
Small for gestational age	34 (12.5)	43 (15.8)	27 (9.9)	27 (9.9)	27 (9.9)	0.13
Large for gestational age	22 (8.1)	19 (7.0)	25 (9.2)	30 (11.0)	24 (8.8)	0.55
Stillbirth	2 (0.7)	2 (0.7)	2 (0.7)	1 (0.4)	0 (0)	0.68
Labour outcomes (%)						
Induction of labour	145 (53.3)	107 (39.2)	92 (33.8)	72 (26.5)	64 (23.4)	< 0.001
Elective caesarean section	47 (17.3)	33 (12.1)	64 (23.5)	70 (25.7)	46 (16.8)	< 0.001
Mode of delivery (in labour)						
Spontaneous vaginal	88 (39.1)	113 (47.1)	136 (65.4)	169 (83.7)	204 (89.9)	< 0.001
Instrumental delivery	55 (24.4)	59 (24.6)	25 (12.0)	14 (6.9)	7 (3.1)	< 0.001
Emergency CS	82 (36.4)	68 (28.3)	47 (22.6)	19 (9.4)	16 (7.0)	< 0.001
Postpartum haemorrhage	57 (21)	39 (14.3)	32 (11.8)	27 (9.9)	23 (8.4)	< 0.001
Apgar < 7 at 5 min	10 (3.7)	5 (1.8)	5 (1.8)	5 (1.8)	2 (0.7)	0.18
NICU admission	29 (10.7)	23 (8.4)	19 (7.0)	22 (8.1)	18 (6.6)	0.45
Neonatal death	0 (0)	1 (0.4)	0 (0)	3 (1.1)	0 (0)	0.07

^a^Women with miscarriage were not included in the analysis of other outcomes

*CS* caesarean section, *NICU* neonatal intensive care unit

In sensitivity analyses, we found that the point estimates for the associations of clinical predictors with uncomplicated pregnancy and birth were broadly similar in the group with maximal data and those who were excluded because of missing data. Additional file 6 describes these results.

## Discussion

Over one-third of this contemporary, multi-ethnic, inner city population of obese pregnant women had an uncomplicated pregnancy and birth. Nevertheless, this also highlights the extent to which obesity is an important risk factor for pregnancy complications. Combining clinical factors and biomarkers, we identified five independent predictors in early pregnancy (at 15^+0^ to 18^+6^ weeks’ gestation) that were associated with an uncomplicated pregnancy and birth, namely multiparity, lower maternal age, SBP and HbA1c levels, and higher adiponectin levels. In combination, these achieved an AUROC of 0.72 (0.68–0.76) for the prediction of an uncomplicated pregnancy and birth. The use of the upper fifth of predicted risk of uncomplicated pregnancy and birth as the screen positive group provided a sensitivity of 38% and a specificity of 89%.

When considering the individual pregnancy complications, the most prevalent during the antenatal period and labour were GDM and emergency caesarean section, respectively. We found that the prevalence of GDM, preeclampsia and other hypertensive disorders, preterm birth, and postpartum haemorrhage decreased and spontaneous vaginal delivery increased across the five groups defined by increasing predicted chance of uncomplicated pregnancy and birth. There was no strong evidence of any difference in miscarriage, antepartum haemorrhage, placental abruption, venous thrombus-embolism, stillbirth, low Apgar score, admission to neonatal intensive care or neonatal death across the five groups, but the numbers in each were few. In general, however, the prevalence of these adverse events was lowest in the group with highest prediction of uncomplicated pregnancy and birth. This suggests that our approach of using one composite outcome is appropriate as those women predicted to have uncomplicated pregnancy and birth were also those shown to have a lower risk for individual adverse outcomes.

To our knowledge, there has been no previous study addressing a composite outcome of uncomplicated pregnancy and birth in obese women. Our previous study explored the factors associated with uncomplicated pregnancy amongst nulliparous women of any BMI [6]. Approximately 61% of nulliparous women unselected for BMI had uncomplicated pregnancies, compared to 38% in the present cohort of women with obesity. Although we would expect lower rates of uncomplicated pregnancy and birth outcomes in obese women, this difference may be partly attributed to the definition of uncomplicated pregnancy and birth, which varied between studies (instrumental delivery and emergency caesarean section were only considered a pregnancy complication in the present study). Despite the different populations, lower maternal age, SBP and BMI were associated with uncomplicated pregnancy in both studies.

Despite being a widely recognised major risk factor for pregnancy complications [1], the recent World Health Organization recommendations on antenatal care as well as current antenatal guidelines in the UK do not provide specific recommendations for the management of pregnancy in obese women [17, 18]. The American College of Obstetrics and Gynaecology has published a relevant practice bulletin, but this provides recommendations for all obese women and does not offer stratification of care in this large group [19]. We have shown that it is possible to identify different risk groups amongst obese women, with good discrimination and calibration. Ultimately, we hope that this approach might help improve the management of obese pregnant women. Women with the highest prediction of uncomplicated pregnancy and birth (those in the upper fifth) had similar levels of risk of most complications to those seen in an unselected obstetric population [12, 20–22], and might benefit from different pathways of care compared to all obese women. In this selected group of women with obesity, the observed prevalence of GDM (16%) was similar to the incidence described in the HAPO study (unselected population) when using the IADPSG criteria (16%) [12] and the prevalence of preeclampsia (3%) was similar to that previously reported in lean women (3%) and lower than previously observed in women with obesity (9%) [21]. Finally, the prevalence of small for gestational age infants (10%) was similar to an unselected population (9–12%) [20].

Since obese women are currently defined as high risk, a higher AUROC for uncomplicated pregnancy than that achieved by the full model (0.72, 0.68–0.76) in this group might be desirable. This also applies to the prediction tool based on clinical factors (AUROC of 0.68, 0.65–0.71). We therefore recommend that the effectiveness of any alternative pathway of care based on these prediction tools should be fully evaluated prior to implementation in clinical practice. Inclusion of other relevant variables, for example, previous obstetric history, weight change from pre-pregnancy to early second trimester or additional biomarkers, may further improve the accuracy of these models. Further studies should also focus on identifying possible causal mechanisms (i.e. biomarkers of good glycaemic control and lower insulin resistance) contributing to uncomplicated pregnancy and birth, which could be amenable to targeted interventions.

Our results have immediate relevance to the choice of place of birth. According to NICE, all women with a BMI ≥ 35 kg/m^2^ should receive recommendation to deliver in an obstetric unit [23]. However, some high risk women still choose to receive care in low risk settings, and therefore clearer direction is needed for shared decision-making. In the Birthplace study, 35% of ‘higher risk’ women who planned a home birth were obese (BMI ≥ 30 kg/m^2^) [24]. In our study, the rate of spontaneous vaginal delivery in obese women in labour with a BMI ≥ 35 kg/m^2^ was 62% (57–67%), similar to that in women with a BMI of 30–35 kg/m^2^ (66%, 61–71%). Importantly, women identified as most likely to have an uncomplicated pregnancy and birth (upper fifth) by our prediction model had a higher rate of spontaneous vaginal delivery of 92% (87–97%) following onset of labour. This figure was markedly similar (90%, 86–94%) in the simpler model including clinical factors alone. In common with our findings, the Birthplace study also identified a subgroup of lower risk women with a BMI ≥ 35 kg/m^2^ in which the risk for obstetric interventions or maternal adverse outcomes was lower than in nulliparous women of normal BMI (21% vs. 53%) [25]. Together, these observations suggest that the current NICE guideline could be modified.

It is generally accepted that obese women should be offered a 75 g OGTT between 24 and 28 weeks’ gestation. Lower risk obese women in this study had a GDM prevalence of 16%, similar to the prevalence in an unselected population with the same IADPSG diagnostic criteria [12]. Therefore, it is difficult to suggest a different screening approach for GDM that would be widely accepted. However, the women least likely to have an uncomplicated pregnancy and birth had a GDM prevalence of 43%. These women are likely to benefit from early management or increased surveillance (i.e. prophylactic interventions or early pregnancy OGTT) [26]. Similarly, higher preeclampsia rates were observed for women least likely to have an uncomplicated pregnancy and birth; therefore, these women may benefit from additional blood pressure measurements.

A key strength of this study lies in the prospective collection of detailed clinical, anthropometric and biomarker data related to obesity with contemporaneous data monitoring across all sites. Another strength lies in the assessment of uncomplicated pregnancy and birth, and in the principle of a prediction tool applicable to all pregnancies in obese women rather than one for each outcome (i.e. GDM, preeclampsia, fetal growth restriction). This novel concept simplifies stratification in obese women who are at increased risk of multiple adverse outcomes. A limitation was the number of women without biomarker data (286/1409, 27%). To explore potential selection bias, we assessed the effect size of each clinical factor in the whole study population and in the restricted study cohort (with available biomarkers); similar effect sizes were observed in both samples. A ‘healthy cohort’ effect cannot be excluded; obese women who took part in UPBEAT may have been healthier than the general obese pregnant population, and our prediction model performance would not be generalisable. However, we consider this unlikely as UPBEAT participants had maternal demographic characteristics usually associated with the poorest pregnancy outcomes, being on average 10 months older and with a BMI 0.7 kg/m^2^ higher compared to women who declined participation [8]. Other limitations include the low prevalence of some outcomes, such as stillbirth or neonatal death, leading to reduced statistical power. We also acknowledge that external independent validation of our prediction tool is necessary.

## Conclusion

Approximately one-third of the obese women studied had an uncomplicated pregnancy and birth. We have shown that risk stratification could be achieved by a combination of clinical factors and biomarkers. Stratification of risk for an uncomplicated pregnancy is an innovative approach to the management of obese pregnant women, with the potential to improve clinical management and the choices for women as well as to ensure efficient resource allocation. Further studies are needed to explore alternative pathways of care for the sub-groups of obese women who differ according to risk profile for uncomplicated pregnancy and birth.

## Additional files

Additional file 1: Analytical methodologies for the biomarkers measured. (DOCX 13 kb)

Additional file 2: Sociodemographic characteristics of study population and UPBEAT participants excluded from this analysis. (DOCX 13 kb)

Additional file 3: Distribution of antenatal and labour complications. (DOCX 38 kb)

Additional file 4: Factors assessed in relation to uncomplicated pregnancy and birth. (DOCX 15 kb)

Additional file 5: Pregnancy outcomes according to predicted chance in fifths of uncomplicated pregnancy and birth using the model with clinical factors and HbA1c. (DOCX 15 kb)

Additional file 6: Sensitivity analysis to assess consistency of association of clinical factors in a restricted sample (women with biomarker data). (DOCX 12 kb)

## Abbreviations

AUROC: area under the receiver operating characteristic
BMI: body mass index
GDM: gestational diabetes mellitus
gGT: gamma-glutamyl transferase
HbA1c: haemoglobin A1c
IADPSG: International Association of Diabetes and Pregnancy Study Groups
OGTT: oral glucose tolerance test
SBP: systolic blood pressure
UPBEAT: UK Pregnancies Better Eating and Activity Trial
