# Supplementary material for: Prediction of uncomplicated pregnancies in obese women: a prospective multicentre study
Source: BMC Med. 2017 Nov 3;15:194. doi: 10.1186/s12916-017-0956-8 (PMC5669007; doi:10.1186/s12916-017-0956-8)
Supplement: Supplementary file 1 — Analytical methodologies for the biomarkers measured. (DOCX 13 kb) [file 12916_2017_956_MOESM1_ESM.docx]

Additional file 1 - Table. Analytical methodologies for the biomarkers measured.

| **Biomarker** | **Sample** | **Method** | **Platform** | **Coefficient of Variation** |
| --- | --- | --- | --- | --- |
| HbA1c | Whole blood | Turbidimetric inhibition immunoassay | Roche, Cobas c311 | low 1.4%, high 1.3% |
| Fructosamine | Plasma | Colorimetric, nitroblue tetrazolium | Roche, Cobas c311 | low 3.4% |
| Insulin | Plasma | Electrochemiluminescence immunoassay | Roche, Cobas e411 | low 7.8%, high 5.4% |
| C-peptide | Serum | Electrochemiluminescence immunoassay | Roche, Cobas e411 | low 6.2%, high 5.1% |
| Adiponectin | Plasma | Enzyme-linked immunosorbent assay | R and D Systems | intra 5.4%, inter 12.0% |
| Leptin | Plasma | Enzyme-linked immunosorbent assay | R and D Systems | intra 2.0%, Inter 9.3% |
| Interleukin-6 | Plasma | Enzyme-linked immunosorbent assay | R and D Systems | intra 9.8%, inter 12.8% |
| C-reactive protein | Plasma | Particle enhanced immunoturbidimetric | Roche, Cobas c311 | low 7.1% |
| tPA antigen | Plasma | Enzyme-linked immunosorbent assay | Asserchrom tpa (Stago) | Intra 5.7%, Inter 7.9% |
| Triglycerides | Plasma | Enzymatic, colorimetric | Roche, Cobas c311 | low 2.2%, high 1.8% |
| Total cholesterol | Plasma | Enzymatic, colorimetric | Roche, Cobas c311 | low 1.8%, high 1.1% |
| LDL cholesterol | Plasma | Homogenous Enzymatic, colorimetric | Roche, Cobas c311 | low 2.0% |
| HDL cholesterol | Plasma | Homogenous Enzymatic, colorimetric | Roche, Cobas c311 | low 2.0% |
| AST | Plasma | Enzymatic, spectrophotometric | Roche, Cobas c311 | low 2.1%, high 1.8% |
| ALT | Plasma | Enzymatic, spectrophotometric | Roche, Cobas c311 | low 3.3%, high 3.1% |
| gGT | Plasma | Enzymatic, colorimetric | Roche, Cobas c311 | low 3.9%, high 3.9% |
| SHBG | Serum | Electrochemiluminescence immunoassay | Roche, Cobas e411 | low 5.9%, high 7.9% |
| Ferritin | Plasma | Particle enhanced immunoturbidimetric | Roche, Cobas c311 | low 1.7% |
| Vitamin D | Serum | Electrochemiluminescence immunoassay | Roche, Cobas e411 | low CV 11.2%, High 9,2% |

Abbreviations: ALT - alanine aminotransferase, AST - aspartate aminotransferase, gGT - gamma-glutamyl transferase, HbA1c - Haemoglobin A1c, and SHBG - sex hormone binding globulin.
