# Supplementary material for: Prediction of uncomplicated pregnancies in obese women: a prospective multicentre study
Source: BMC Med. 2017 Nov 3;15:194. doi: 10.1186/s12916-017-0956-8 (PMC5669007; doi:10.1186/s12916-017-0956-8)
Supplement: Supplementary file 2 — Sociodemographic characteristics of study population and UPBEAT participants excluded from this analysis. (DOCX 13 kb) [file 12916_2017_956_MOESM2_ESM.docx]

Additional file 2 - Table. Socio-demographic characteristics of study population and UPBEAT participants excluded from this analysis.

|  | **Study population** | **Women excluded** |  |
| --- | --- | --- | --- |
|  | **Mean (SD) or n (%)** | **Mean (SD) or n (%)** | **p value** |
| Age | 30.4 (5.5) | 31.1 (5.4) | 0.16 |
| Body mass index | 36.4 (4.8) | 35.8 (4.4) | 0.19 |
| Ethnicity |  |  |  |
| White | 886/1,409 (62.9) | 78/129 (60.5) |  |
| Black | 356/1,409 (25.3) | 39/129 (30.2) | 0.57 |
| Asian | 86/1,409 (6.1) | 8/129 (6.2) |  |
| Other | 81/1,409 (5.7) | 4/129 (3.1) |  |
| Multiparous | 786/1,409 (44.2) | 82/129 (36.4) | 0.09 |
| Previous history of GDM | 28/786 (3.6) | 4/82 (4.9) | 0.55 |
| Previous history of PE | 62/786 (7.9) | 7/82 (8.5) | 0.84 |
| Family history |  |  |  |
| Diabetes (type2) | 328/1,406 (23.3) | 40/129 (31) | 0.05 |
| Hypertension | 642/1,406 (45.7) | 70/129 (54.3) | 0.06 |
| IMD fifths |  |  |  |
| 1 (least deprived) | 52/1,403 (3.7) | 13/129 (10.1) |  |
| 2 | 94/1,403 (6.7) | 7/129 (5.4) |  |
| 3 | 156/1,403 (11.1) | 17/129 (13.2) | 0.18 |
| 4 | 480/1,403 (34.2) | 47/129 (36.4) |  |
| 5 (most deprived) | 621/1,403 (44.3) | 45/129 (34.9) |  |
| Current smoker | 98/1,409 (7) | 8/129 (6.2) | 0.75 |

Abbreviations: GDM - gestational diabetes mellitus, IMD – index of multiple deprivation, and PE – pre-eclampsia
