# Supplementary figures and images for: Prediction of uncomplicated pregnancies in obese women: a prospective multicentre study
Source: BMC Med. 2017 Nov 3;15:194. doi: 10.1186/s12916-017-0956-8 (PMC5669007; doi:10.1186/s12916-017-0956-8)

Additional file 3 - Figure. Distribution of antenatal and labour complication.


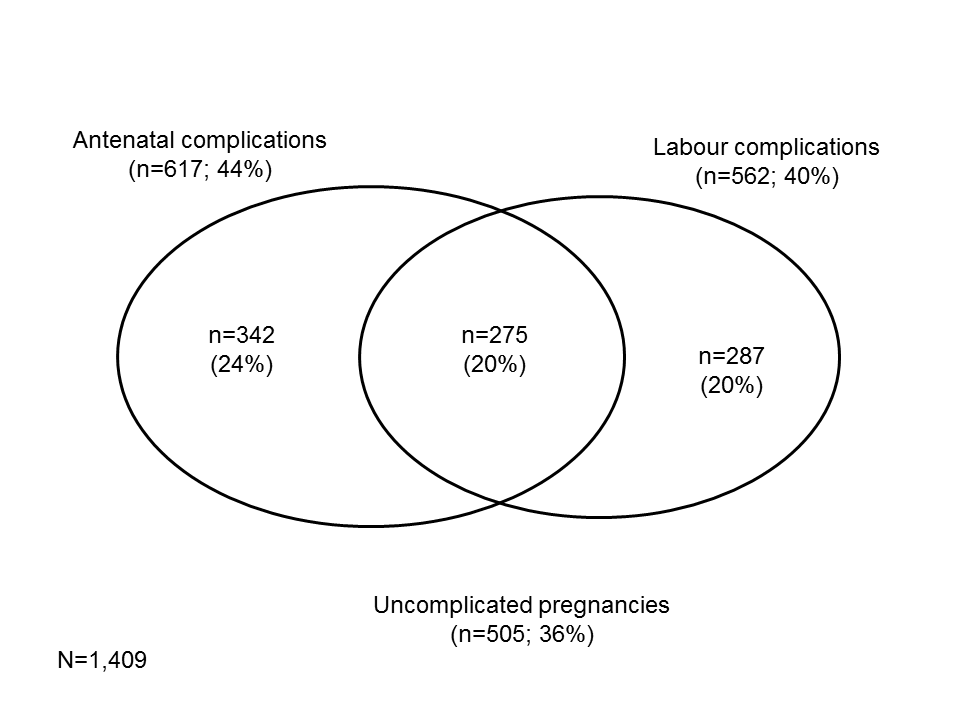

Supplement: Supplementary file 3 — Distribution of antenatal and labour complications. (DOCX 38 kb) [file 12916_2017_956_MOESM3_ESM.docx]
