# Supplementary material for: Prediction of uncomplicated pregnancies in obese women: a prospective multicentre study
Source: BMC Med. 2017 Nov 3;15:194. doi: 10.1186/s12916-017-0956-8 (PMC5669007; doi:10.1186/s12916-017-0956-8)
Supplement: Supplementary file 4 — Factors assessed in relation to uncomplicated pregnancy and birth. (DOCX 15 kb) [file 12916_2017_956_MOESM4_ESM.docx]

Additional file 4 - Table. Factors assessed in relation to uncomplicated pregnancy and birth.

|  | **Complicated** | **Uncomplicated** |  |  |
| --- | --- | --- | --- | --- |
|  | **Mean (SD) or n (%)** | **Mean (SD) or n (%)** | **p value** |  |
| Age (years) | 30.6 (5.4) | 30.0 (5.7) | 0.04 |  |
| White ethnicity | 567/904 (62.7) | 319/505 (63.2) | 0.87 |  |
| Index of multiple deprivation (fifths) |  |  |  |  |
| 1 (least deprived) | 33/899 (3.7) | 19/504 (3.8) |  |  |
| 2 | 62/899 (6.9) | 32/504 (6.4) |  |  |
| 3 | 95/899 (10.6) | 61/504 (12.1) | 0.80 |  |
| 4 | 316/899 (35.2) | 164/504 (32.5) |  |  |
| 5 (most deprived) | 393/899 (43.7) | 228/504 (45.2) |  |  |
| Body mass index (kg/m^2^) | 36.6 (5.2) | 35.9 (4.1) | 0.003 |  |
| Multiparity | 416/904 (46) | 370/505 (73.3) | <0.001 |  |
| Previous history of GDM or PE | 57/416 (13.7) | 31/370 (8.4) | 0.02 |  |
| Smoking | 66/904 (7.3) | 32/505 (6.3) | 0.50 |  |
| Threatened miscarriage | 211/903 (23.4) | 101/501 (20.2) | 0.17 |  |
| Systolic blood pressure (mmHg) | 118.7 (11.0) | 115.6 (10.6) | <0.001 |  |
| Mid arm circumference (cm) | 37.1 (4.3) | 36.4 (3.8) | 0.005 |  |
| Sum of skinfolds (mm) | 124.7 (28.2) | 120.4 (26.1) | 0.005 |  |
| **Biomarkers** |  |  |  |  |
| HbA1c (mmol/mol) | | 30.0 (3.9) | 28.6 (3.5) | <0.001 |
| Fructosamine (umol/l) | | 187.0 (21.1) | 186.3 (20.8) | 0.62 |
| Insulin (mU/l), log_2_ | | 4.8 (1.4) | 4.6 (1.4) | 0.002 |
| C-peptide (ng/ml), log_2_ | | 2.1 (0.8) | 1.9 (0.8) | 0.03 |
| Adiponectin (ug/ml), log_2_ | | 3.2 (0.9) | 3.5 (0.9) | <0.001 |
| Leptin (pg/ml), log_2_ | | 6.0 (0.7) | 6.0 (0.6) | 0.12 |
| Interleukin-6 (pg/ml), log_2_ | | 1.6 (0.9) | 1.5 (0.9) | 0.17 |
| C-reactive protein (mg/l), log_2_ | | 2.7 (1.1) | 2.6 (1.1) | 0.84 |
| t-PA antigen (ng/ml), log_2_ | | 2.8 (0.8) | 2.7 (0.7) | 0.04 |
| Triglycerides (mmol/l), log_2_ | | 0.8 (0.5) | 0.7 (0.5) | 0.05 |
| Total cholesterol (mmol/l) | | 5.7 (1.0) | 5.7 (1.0) | 0.55 |
| LDL cholesterol (mmol/l) | | 2.8 (0.8) | 2.8 (0.8) | 0.30 |
| HDL cholesterol (mmol/l), log_2_ | | 0.6 (0.5) | 0.6 (0.4) | 0.61 |
| AST (U/l), log_2_ | | 4.5 (0.5) | 4.5 (0.5) | 0.12 |
| ALT (U/l), log_2_ | | 4.2 (0.8) | 4.1 (0.7) | 0.02 |
| gGT (U/l), log_2_ | | 3.8 (1.0) | 3.6 (0.9) | <0.001 |
| SHBG (nmol/l) | | 409.3 (120.8) | 438.8 (131.4) | <0.001 |
| Ferritin (ng/ml), log_2_ | | 5.6 (1.1) | 5.4 (1.1) | 0.02 |
| Vitamin D (ng/ml), log_2_ | | 3.8 (0.9) | 3.9 (0.9) | 0.46 |

Abbreviations: ALT - alanine aminotransferase, AST - aspartate aminotransferase, GDM - gestational diabetes mellitus, gGT - gamma-glutamyl transferase, HbA1c - Haemoglobin A1c, PE – pre-eclampsia, and SHBG - sex hormone binding globulin.
