# Supplementary material for: Prediction of uncomplicated pregnancies in obese women: a prospective multicentre study
Source: BMC Med. 2017 Nov 3;15:194. doi: 10.1186/s12916-017-0956-8 (PMC5669007; doi:10.1186/s12916-017-0956-8)
Supplement: Supplementary file 5 — Pregnancy outcomes according to predicted chance in fifths of uncomplicated pregnancy and birth using the model with clinical factors and HbA1c. (DOCX 15 kb) [file 12916_2017_956_MOESM5_ESM.docx]

Additional file 5 - Table. Pregnancy outcomes according to predicted chance in fifths of uncomplicated pregnancy and birth using the model with clinical factors and HbA1c.

|  | **Least likely to have an uncomplicated pregnancy and birth** | |  | **Most likely to have an uncomplicated pregnancy and birth** | |  |
| --- | --- | --- | --- | --- | --- | --- |
|  | **1^st^ fifth** | **2^nd^ fifth** | **3^rd^ fifth** | **4^th^ fifth** | **5^th^ fifth** |  |
|  | **(n=181)** | **(n=181)** | **(n=182)** | **(n=181)** | **(n=182)** | **p value** |
| **Uncomplicated pregnancy (%)** | 20 (11.0) | 38 (21.0) | 60 (33.0) | 82 (45.3) | 103 (56.6) | <0.001 |
| **Antenatal Outcomes (%)** |  |  |  |  |  |  |
| Miscarriage ^a^ | 3 (1.7) | 2 (1.1) | 1 (0.5) | 1 (0.6) | 1 (0.5) | 0.73 |
| Preterm delivery | 10 (5.6) | 9 (5.0) | 15 (8.3) | 3 (1.7) | 8 (4.4) | 0.07 |
| Gestational diabetes | 74 (41.6) | 51 (28.5) | 49 (27.1) | 40 (22.2) | 34 (18.8) | <0.001 |
| Preeclampsia | 16 (9.0) | 7 (3.9) | 8 (4.4) | 3 (1.7) | 6 (3.3) | 0.01 |
| Other hypertensive disorders | 18 (10.1) | 11 (6.1) | 12 (6.6) | 10 (5.6) | 1 (0.6) | 0.004 |
| Antepartum haemorrhage | 9 (5.1) | 8 (4.5) | 6 (3.3) | 10 (5.6) | 5 (2.8) | 0.66 |
| Placental abruption | 1 (0.6) | 1 (0.6) | 1 (0.6) | 2 (1.1) | 1 (0.6) | 0.96 |
| Venous thromboembolism | 0 (0) | 1 (0.6) | 0 (0) | 0 (0) | 0 (0) | 0.40 |
| Small for gestational age | 26 (14.6) | 30 (16.8) | 17 (9.4) | 23 (12.8) | 15 (8.3) | 0.08 |
| Large for gestational age | 16 (9.0) | 12 (6.7) | 19 (10.5) | 14 (7.8) | 19 (10.5) | 0.65 |
| Stillbirth | 2 (1.1) | 0 (0) | 1 (0.6) | 2 (1.1) | 0 (0) | 0.40 |
| **Labour Outcomes (%)** |  |  |  |  |  |  |
| Induction of labour | 92 (51.7) | 77 (43.0) | 70 (38.7) | 41 (22.8) | 45 (24.9) | <0.001 |
| Elective caesarean section | 37 (20.8) | 24 (13.4) | 35 (19.3) | 45 (25.0) | 41 (22.7) | 0.08 |
| Mode of delivery (in labour) |  |  |  |  |  |  |
| Spontaneous vaginal | 52 (36.9) | 77 (49.7) | 94 (64.4) | 112 (83.0) | 123 (87.9) | <0.001 |
| Instrumental delivery | 25 (17.7) | 38 (24.5) | 19 (13.0) | 14 (10.4) | 6 (4.3) | <0.001 |
| Emergency CS | 64 (45.4) | 40 (25.8) | 33 (22.6) | 9 (6.7) | 11 (7.9) | <0.001 |
| Postpartum haemorrhage | 48 (27.0) | 31 (17.3) | 19 (10.5) | 22 (12.2) | 15 (8.3) | <0.001 |
| Apgar<7 at 5th minute | 6 (3.4) | 4 (2.2) | 3 (1.7) | 3 (1.7) | 3 (1.7) | 0.75 |
| NICU admission | 18 (10.1) | 20 (11.2) | 18 (9.9) | 7 (3.9) | 13 (7.2) | 0.09 |
| Neonatal death | 0 (0) | 2 (1.1) | 0 (0) | 0 (0) | 1 (0.6) | 0.25 |

Abbreviations: CS – caesarean section, and NICU - neonatal intensive care unit.

^a^ Women with miscarriage were not included in the analysis of other outcomes.
