# Supplementary material for: Prediction of uncomplicated pregnancies in obese women: a prospective multicentre study
Source: BMC Med. 2017 Nov 3;15:194. doi: 10.1186/s12916-017-0956-8 (PMC5669007; doi:10.1186/s12916-017-0956-8)
Supplement: Supplementary file 6 — Sensitivity analysis to assess consistency of association of clinical factors in a restricted sample (women with biomarker data). (DOCX 12 kb) [file 12916_2017_956_MOESM6_ESM.docx]

Additional file 6 - Table. Sensitivity analysis to assess consistency of association of clinical factors in a restricted sample (women with biomarker data).

|  | **Study population** | **Restricted sample ^a^** |
| --- | --- | --- |
|  | **OR (95%CI)** | **OR (95%CI)** |
| Maternal age (per 5 year) | 0.79 (0.71 - 0.88) | 0.78 (0.68 - 0.90) |
| Multiparous | 3.54 (2.75 - 4.55) | 3.66 (2.67 – 5.02) |
| Systolic Blood Pressure (per 10 mmHg) | 0.82 (0.73 - 0.91) | 0.86 (0.75 - 0.98) |
| Body Mass Index (per 5 kg/m2) | 0.85 (0.74 - 0.97) | 0.82 (0.70 - 0.97) |

^a^ Participants of the multivariable model including clinical factors and biomarkers (n=907)
